# CD28 Down-Regulation on Circulating CD4 T-Cells Is Associated with Poor Prognoses of Patients with Idiopathic Pulmonary Fibrosis

**DOI:** 10.1371/journal.pone.0008959

**Published:** 2010-01-29

**Authors:** Syed R. Gilani, Louis J. Vuga, Kathleen O. Lindell, Kevin F. Gibson, Jianmin Xue, Naftali Kaminski, Vincent G. Valentine, Emily K. Lindsay, M. Patricia George, Chad Steele, Steven R. Duncan

**Affiliations:** 1 Department of Medicine, University of Pittsburgh, Pittsburgh, Pennsylvania, United States of America; 2 Department of Medicine, University of Texas Medical Branch, Galveston, Texas, United States of America; 3 Department of Medicine, University of Alabama, Birmingham, Alabama, United States of America; University of California Los Angeles, United States of America

## Abstract

**Background:**

Although the etiology of idiopathic pulmonary fibrosis (IPF) remains perplexing, adaptive immune activation is evident among many afflicted patients. Repeated cycles of antigen-induced proliferation cause T-cells to lose surface expression of CD28, and we hypothesized this process might also occur in IPF.

**Methodology/Principal Findings:**

Peripheral blood CD4 T-cells from 89 IPF patients were analyzed by flow cytometry and cytokine multiplex assays, and correlated with clinical events. In comparison to autologous CD4^+^CD28^+^cells, the unusual CD4^+^CD28^null^ lymphocytes seen in many IPF patients had discordant expressions of activation markers, more frequently produced cytotoxic mediators perforin (2.4±0.8% vs. 60.0±7.4%, p<0.0001) and granzyme B (4.5±2.8% vs.74.9±6.5%, p<0.0001), produced greater amounts of many pro-inflammatory cytokines, and less frequently expressed the regulatory T-cell marker FoxP3 (12.9±1.1% vs. 3.3±0.6% p<0.0001). Infiltration of CD4^+^CD28^null^ T-cells in IPF lungs was confirmed by confocal microscopy. Interval changes of CD28 expression among subjects who had replicate studies were correlated with conterminous changes of their forced vital capacities (*r_s_* = 0.49, p = 0.012). Most importantly, one-year freedom from major adverse clinical events (either death or lung transplantation) was 56±6% among 78 IPF patients with CD4^+^CD28^+^/CD4_total_≥82%, compared to 9±9% among those with more extensive CD28 down-regulation (CD4^+^CD28^+^/CD4_total_<82%) (p = 0.0004). The odds ratio for major adverse events among those with the most extensive CD28 down-regulation was 13.0, with 95% confidence intervals 1.6-111.1.

**Conclusions/Significance:**

Marked down-regulation of CD28 on circulating CD4 T-cells, a result of repeated antigen-driven proliferations, is associated with poor outcomes in IPF patients. The CD4^+^CD28^null^ cells of these patients have potentially enhanced pathogenic characteristics, including increased productions of cytotoxic mediators and pro-inflammatory cytokines. These findings show proliferative T-cell responses to antigen(s) resulting in CD28 down-regulation are associated with progression and manifestations of IPF, and suggest assays of circulating CD4 T-cells may identify patients at greatest risk for clinical deterioration.

## Introduction

Idiopathic pulmonary fibrosis (IPF) is a chronic fibroproliferative lung disease that afflicts ∼40,000 patients in the U.S. each year [1]. IPF typically manifests with inexorable pulmonary restriction and hypoxemia, resulting in progressive exercise limitation and dyspnea. The disease has a grim prognosis, with a median survival of ∼3 years after diagnosis, although courses can be highly variable. No medical treatments have yet been shown to alter the natural history of this disease [1], [2].

The etiology of IPF is enigmatic. Although not widely considered to be an immunologic disorder [2], several studies have shown that adaptive immune responses are often active in IPF patients, as evidenced by the frequent presence of IgG autoantibodies, increased productions of lymphocyte-derived inflammatory mediators, and abnormal extents of T-cell activation and clonal proliferations [3]–[13]. Activated CD4 T-cells may also infiltrate into IPF lungs prior to development of symptoms [14], and pro-inflammatory dendritic cells accumulate in the pulmonary parenchyma of advanced cases [15]. FoxP3^+^ regulatory T-cells (T_reg_), widely regarded as having important roles in dampening adaptive immune responses against antigens [16], are diminished in both the circulation and broncholavolar lavage returns of IPF patients [17]. More directly compelling, a protein(s) uniquely present in IPF lungs induces proliferation of autologous CD4 T-cells from these patients [13].

We hypothesized that if IPF progression does indeed have an important adaptive immune component, we may be able to find associations between phenotypic alterations of circulating T-cells and clinical manifestations of the disease. We focused attention on CD4 T-lymphocytes of the patients, given the singular role of these cells in orchestrating adaptive immune responses [18], and their responsiveness to IPF-associated intrapulmonary antigen(s) [13]. Nearly all CD4 T-cells of healthy, normal individuals express CD28, a co-stimulatory molecule that provides a “second signal” for activation of naïve CD4 lymphocytes. However, human T-cells that have undergone repeated cycles of antigen-driven proliferation develop a series of phenotypic and functional changes, including the disappearance of cell-surface CD28. Hence, finding significant proportions of circulating CD4^+^CD28^null^ cells is a *de facto* marker denoting the presence of a chronic adaptive immune response [19]–[34].

Accordingly, we characterized CD28 expression on the circulating CD4 T-cells of a well-defined IPF cohort, and correlated results of the immunologic assays with clinical features of these patients. We found abnormally large proportions of peripheral CD4 T-cells in some IPF patients lack CD28 expression, and these CD4^+^CD28^null^ cells also have unusual, and potentially pathogenic, functional alterations. Most importantly, the presence of significant CD28 down-regulation identified IPF subjects who had particularly poor clinical outcomes, analogous to observations in other patient populations with chronic immunologic diseases [20]–[23], [32]–[34].

## Results

### CD4 T-Cell Expression of CD28 among IPF Patients

**Figure 1:**
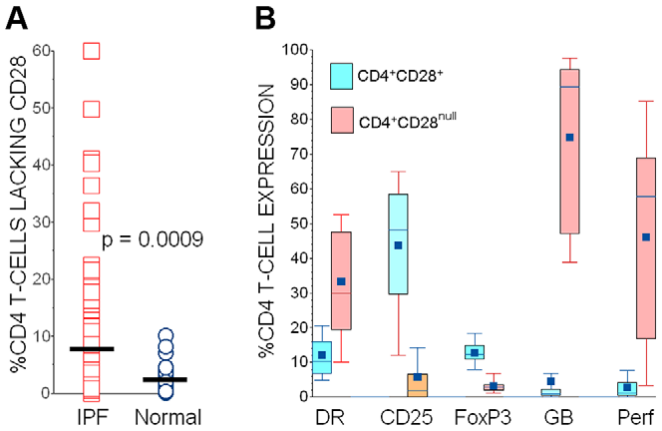
Characteristics of CD4 T-cell subpopulations in IPF patients. *A:* The proportions of circulating CD4 T-cells that also expressed CD28 (CD28%) were reduced in many IPF patients. The horizontal line denotes the population means. *B:* In contrast to autologous CD4^+^CD28^+^ cells, the CD4^+^CD28^null^ T-cells of IPF patients more often express major histocompatibility antigen (MHC) Class II (DR), but less frequently express CD25. CD4^+^CD28^null^ T-cells of IPF patients less frequently produce transcription factor FoxP3 (a putative marker of regulatory T-cells), but much more frequently produce cytotoxic mediators granzyme B (GB) and perforin (Perf). For each measure n = 24, and p values for all intergroup comparisons (CD4^+^CD28^+^ vrs. CD4^+^CD28^null^ cells) are <0.0001.

Eighty-nine (89) IPF subjects had one or more assays of their peripheral blood CD4 T-cells. Unless otherwise specified, only results of their latest determinations are described. Clinical observation periods following their last experimental T-cell assays exceeded 12 months in all subjects who survived and/or did not have lung transplantation. The proportion of total circulating CD4 T-cells that co-expressed CD28 were determined for each subject, and these values are defined here as CD28% [20]. CD28 down-regulation was striking in some IPF patients (Figure 1A), and nineteen (21%) of these subjects had CD28% <90. Ages of the 32 normal, healthy controls (67.9±1.1 years old) were comparable to those of the IPF subjects (p = N.S.). None of the normal controls had CD28% <90, and their aggregate values of this parameter (97.9±0.4) were significantly greater than those of the IPF subjects (Figure 1A).

### Phenotypic Features of IPF CD4^+^CD28^null^ Cells

We performed a series of assays (Figure 1B) to ascertain if the CD4^+^CD28^null^ cells of IPF patients exhibited unusual phenotype characteristics, as has been observed elsewhere in comparable cells from patients with chronic adaptive immune disorders [19]–[34].

Because the CD4^+^CD28^null^ lymphocytes are daughter progeny of repetitive, antigen-driven T-cell replication cycles [19], [20], [25], [27], [30], [31], we anticipated these cells would be highly activated. A greater proportion of the IPF CD4^+^CD28^null^ did express major histocompatibility antigen (MHC) Class II (HLA-DR) than the corresponding autologous CD4^+^CD28^+^ cells. However, IPF CD4^+^CD28^null^ cells paradoxically expressed less CD25 than autologous CD4^+^CD28^+^ cells, and this discordant expression of activation markers has also been previously reported in cells derived from another patient population [20]. There was no apparent correlation between CD4 values (as percentages of total peripheral blood mononuclear cells [PBMNC]) and CD28% (*r_s_* = 0.16, p = N.S.)

Selected intracellular factors and mediators with potential clinical relevance were also evaluated here. The lesser expression of CD25 by IPF CD4^+^CD28^null^ cells prompted us to measure FoxP3, and this transcription factor and marker for regulatory T-cell (T_reg_) function was present in significantly fewer of these cells than among corresponding CD4^+^CD28^+^ lymphocytes (Figure 1B).

Pulmonary epithelial cell death may be an important pathologic process in IPF [35], leading us to also evaluate potential cytotoxic functions of the CD4 T-cell subpopulations. Cell surface expression of FasL was at or below the detection threshold for flow cytometry in both CD4 subpopulations (data not shown), but the IPF CD4^+^CD28^null^ lymphocytes frequently produced perforin and granzyme B, in striking contrast to autologous CD4^+^CD28^+^ cells (Figure 1B).

### CD4^+^CD28^null^ T-Cells of IPF Patients Produce Pro-Inflammatory Cytokines

**Figure 2:**
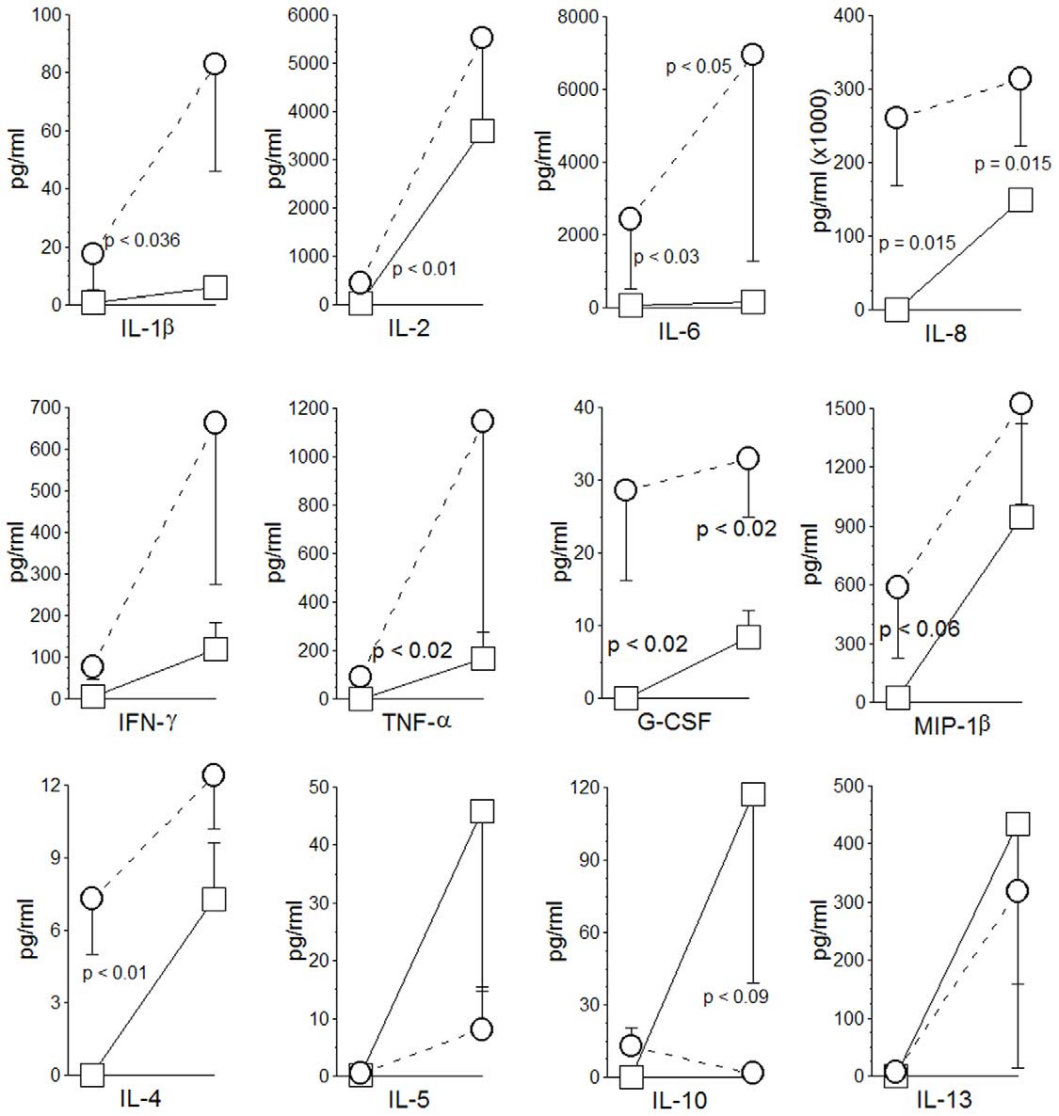
Cytokine elaborations by autologous CD4 subpopulations of IPF patients. Initial (left) data point in each series represents control unstimulated (basal) condition, while second (right) data point delineates productions of cells after stimulation with plate bound anti-CD3 antibody. These paired specimens (control and stimulated) are also connected by lines. CD4^+^CD28^null^ cells from IPF patients (open circles with paired specimens connected by dashed lines) tend to elaborate greater amounts of pro-inflammatory and T_H_1 cytokines (top two rows), whereas CD4^+^CD28^+^ cells (open squares with paired specimens connected by solid lines) have an apparent T_H_2 bias, with the exception of IL-4 production (bottom row) (n = 6 randomly-selected specimens in each measure).

CD4^+^CD28^null^ T-cells from patients with autoimmune diseases or other chronic immunologic disorders have abnormal productions of various cytokines that may have potential importance in disease pathogenesis [19], [20], [24]. Differential cytokine productions of autologous CD4^+^CD28^+^ and CD4^+^CD28^null^ cells from these IPF patients were also evident in multiplex analyses (Figure 2), and the mediator elaboration profiles of these lymphocytes were similar to those of comparable cell subpopulations isolated from transplantation recipients [20]. CD4^+^CD28^null^ cells generally produced much greater amounts of pro-inflammatory and T_H_1 mediators under basal conditions, and in many cases these productions were strikingly increased by T-cell antigen receptor (TCR) cross-linking with anti-CD3 antibody (thereby replicating effects of T-cell-antigen engagements) (Figure 2). Conversely, and with the exception of IL-4, productions of T_H_2 cytokines, notably including putatively immunosuppressive IL-10, were reduced among the CD4^+^CD28^null^ cells in comparisons to autologous CD4^+^CD28^+^cells.

### CD4^+^CD28^null^ Lymphocytes Isolated from IPF Patients Are Capable of Further Proliferation

Several studies of CD4^+^CD28^null^ cell lines derived by extensive *ex vivo* propagation suggest these lymphocytes are replicatively sencescent [19]. Nevertheless, we have previously shown that freshly isolated CD4^+^CD28^null^ lymphocytes from lung transplant recipients are able to proliferate with TCR cross-linking [20], raising the possibility that the reported inability of CD4^+^CD28^null^ cell lines to divide is a consequence of their protracted *in vitro* propagation. In order to further evaluate this hypothesis, we measured proliferations of segregated CD4^+^CD28^+^ and CD4^+^CD28^null^ lymphocytes from IPF subjects after stimulation that mimics T-cell engagement with antigen-MHC complexes (e.g., plate bound anti-CD3 monoclonal antibodies).

**Figure 3:**
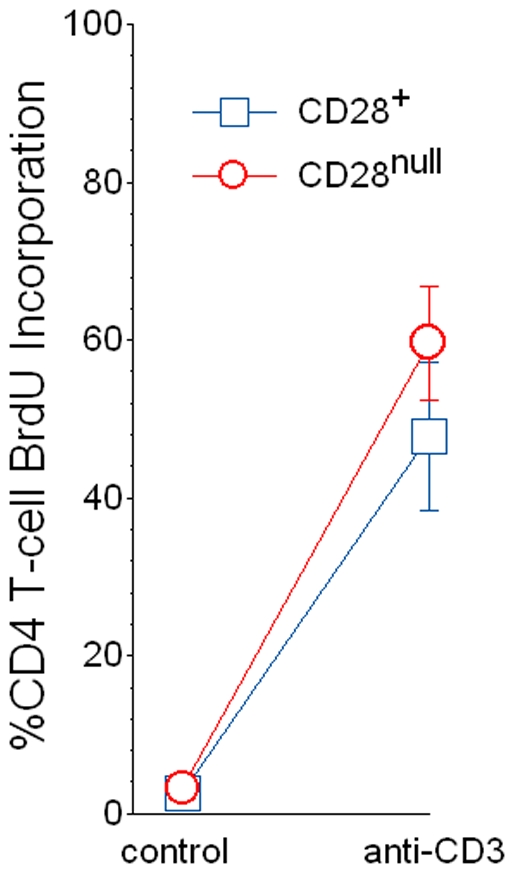
Segregated autologous CD4^+^CD28^+^ and CD4^+^CD28^null^ cells from peripheral blood of IPF patients (n = 9) had similar proliferations (determined by BrdU incorporation) in 5-day control (unstimulated) cultures, as well as after stimulation with plate-bound anti-CD3 monoclonal antibody.

As was seen in previous study of cells isolated from allograft recipients [20], proliferation of CD4^+^CD28^null^ T-cells from the IPF patients was near identical to that of autologous CD4^+^CD28^+^ cells (Figure 3).

### CD28 Expression Is Associated with Changes of Pulmonary Function in IPF Patients

Pulmonary function tests (PFT) were ordered by physicians caring for these patients on the basis of clinical indications, while blinded to the experimental immunologic assays. Seventy-one (71) subjects had CD4 T-cell assays concurrent with measures of forced vital capacity (FVC), and 57 of these also had determinations of diffusing capacity for carbon monoxide (DLCO). CD28% values modestly, but significantly correlated with concordant DLCO of the subjects (*r_s_* = 0.34, p = 0.01). The cross-sectional relationships between CD28% values and other PFT parameters were not significant (data not shown).

Although single, essentially random measures of CD28% had only limited correlation with concurrent PFT values, these particular T-cell assays have been shown elsewhere to be predictive for subsequent changes of physiologic measures [20]. Too many of the subjects here succumbed or had lung transplantations soon after their last experimental T-cell assays, without having had additional, interval PFTs, to render these analyses meaningful. However, 27 study subjects had prior CD28% determinations, along with concurrent PFTs, as participants of an ongoing longitudinal study.

**Figure 4:**
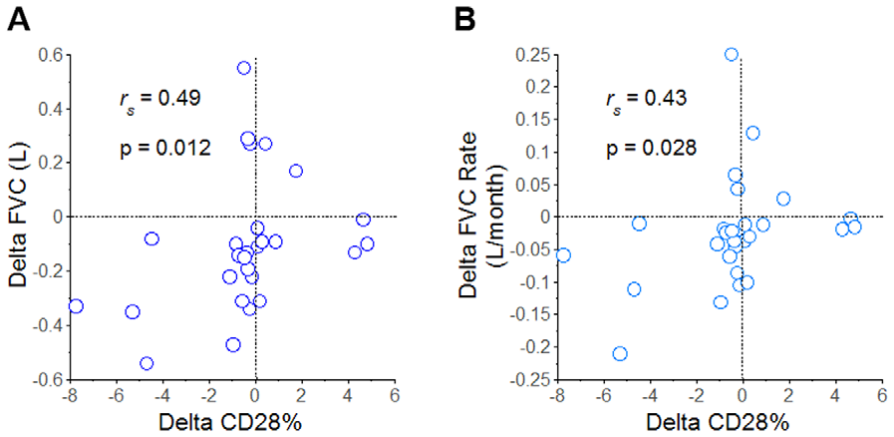
Associations of CD28 expression with pulmonary function. Changes of CD28% with replicate testing (Delta CD28%) were significantly correlated with concomittant interval changes of *A.)* absolute FVC (Delta FVC) and *B.)* FVC as a time-dependent rate (Delta FVC/month).

The net change of aggregate CD28% between the preceding and latest determinations among this subpopulation was −0.44±0.53. Ten (10) of these patients had increases of CD28% (1.7±0.6) with repeat testing 4.6±0.6 months later, in contrast to the other 17 IPF subjects with interval CD28% decrements (−1.7±0.6) over 4.6±0.5 months. The FVC declined significantly less among those subjects with interval CD28% increases (−0.04±0.05 L) compared to those with decreasing CD28% values (−0.15±0.07 L) (p = 0.05). Moreover, interval changes of CD28% among individual subjects were also correlated with corresponding changes of their lung volumes, in terms of both absolute measures (Figure 4A), and as time-dependent rates (Figure 4B). Although there were also similar trends for greater decrements of DLCO and DLCO%p among the subpopulation with interval decreases of CD28%, these intergroup comparisons did not reach statistical significance (data not shown).

### CD28 Down-Regulation in IPF Patients Is Associated with Clinical Outcomes

Given that CD28% values have also been shown to associate with outcome measures in other populations with chronic immunologic disorders [20]–[23], [32]–[34], we tested the hypothesis that these quantitative CD4 T-cell phenotype assays might have clinical prognostic value for patients with IPF.

The eventual progression of lung dysfunction in IPF patients almost inevitably results in either lung transplantation or the death of those afflicted [1]. However, these dichotomous outcomes are not truly independent events. Lung transplantation ostensibly, albeit by no means invariably, diminishes or delays deaths of IPF patients, although the procedure itself is also associated with a finite inherent mortality [36]. Hence, we initially tallied occurrences of either lung transplantations or deaths as equivalent outcome end-points. Receiver operating characteristic (ROC) curve analyses indicated that cut-off values for CD28% = 82 seemed to provide the best compromise of optimal specificity (the most important consideration) and sensitivity for these major, adverse clinical events.

**Figure 5:**
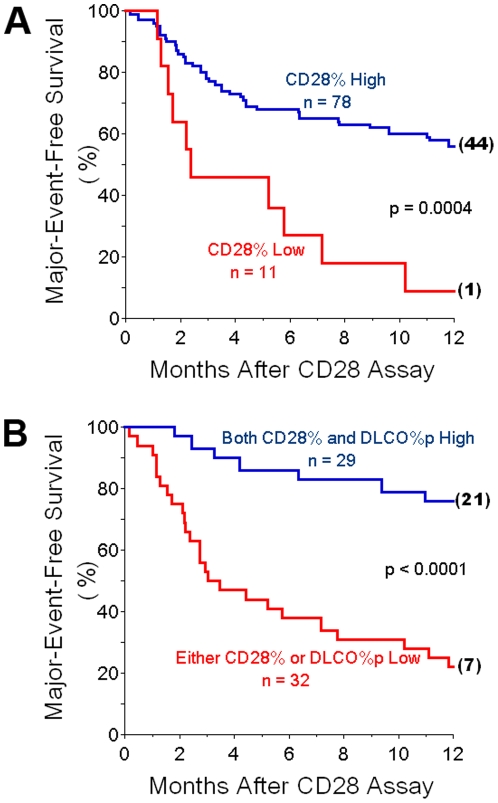
Associations of CD28% espression with clinical outcome. *A.)* Survival curves show cumulative freedom from major adverse events (lung transplantation or death) of IPF patients. Those subjects with the most extreme CD28 down-regulation, with CD28 expressed on <82% of their circulating CD4 T-cells (CD28% Low), had much worse outcomes than the cohort with greater proportions of CD4-Tcells that expressed CD28 (CD28% High). Numbers in parenthesis at the ends of survival curves denote remaining, unafflicted subjects that were censored at 12 months of observation. *B.)* Survival curves showing that cummulative freedom from major adverse events of IPF patients who have either significant CD28 downregulation (CD28% Low) or diffusing capacities for carbon monoxide, as percentages of predicted normal values (DLCO%p) <38, had worse outcomes than the IPF cohort who were both CD28% High and had more normal DLCO%p.

Those IPF patients having the most extreme CD28 down-regulation, with CD28% <82 (henceforth denoted as CD28% Low), had significantly worse 12-month outcomes than the cohort with CD28%≥82 (CD28% High) (Figure 5A). These intergroup survival differences were also apparent with six-month analyses (27% vs. 68%, CD28% Low and CD28% High, respectively, p = 0.004).

Deaths accounted for 24 of the major adverse events, and 20 other subjects had lung transplantations. Seventeen (17) deaths in the study population were directly attributable to respiratory failure. One of the CD28% High subjects died from a myocardial infarction, and another had fatal pulmonary thromboemboli. Five (5) other deaths (four CD28% High and one CD28% Low patient), were due to unknown causes in subjects with preexistent severe pulmonary dysfunction.

To exclude cryptic confounding by potential vagaries of the transplantation selection process (despite “double blinding” between the clinicians and laboratory investigators), we performed *post hoc* actuarial analysis limited to those IPF subjects who did not have lung transplantations during the year after their T-cell assays. Despite lesser statistical power due to fewer subjects, the 12-month survival of the CD28% High patients (68±6%) was still significantly greater than that of the CD28% Low cohort (20±18%) (p = 0.007).

CD28% High and CD28% Low cohorts were well matched with respect to demographic characteristics (Table 1). CD4^+^CD28^null^ cells can reportedly accumulate with aging, but this typically occurs during the eight decade of life or later [19], [25]. Nonetheless, there was no apparent correlation between age and CD28 expression in the IPF cohort (*r_s_* = −0.1, p = 0.36), the overall age distributions of the comparison populations here were equivalent (Table 1), and both the number (n = 15) and proportion (19%) of octogenarians among CD28% High were actually greater than among the CD28% Low (n = 1 [9%]). Absolute DLCO values were decreased in the CD28% Low, but there was a less significant intergroup difference of the DLCO as a percentage of normal predicted values (DLCO%p) (Table 1).

**Table 1:** Subject Characteristics.

	(CD28% High)	(CD28% Low)
**n**	78	11
**Age (years)**	69.6±1.6	71.7±2.6
**Male **–n (%)	55 (71)	8 (73)
**Former Smoker **–n (%)	56 (72)	8 (73)
**Caucasian** –n (%)	76 (97)	10 (91)
**African-American** –n (%)	1 (1)	1 (9)
**Native American** –n (%)	1 (1)	0
**Lung Biopsy**- n (%)	48 (62)	7 (64)
**CD28%**	96.4±0.5	66.2±4.0*
**FVC** (L)(n)	2.4±0.1(62)	2.1±0.2(9)
**FVC%p**(n)	60.5±2.4(62)	57.7±4.3(9)
**FEV_1_/FVC**(n)	0.84±0.01(62)	0.85±0.01(9)
**DLCO** (ml CO/min/mm Hg)(n)	10.7±0.7(50)	6.3±0.9**(7)
**DLCO%p**(n)	43.6±2.3(50)	31.0±4.7†(7)
**Medications**-n (%)		
None	56 (72)	9 (82)
Prednisone	6 (8)	1 (9)
IFN-γ	2 (3)	0
IFN-γ + Prednisone	7 (9)	0

CD28% High denotes those subjects in whom ≥82% of their circulating CD4 T-cells co-express CD28. CD28% Low denotes those subjects in whom <82% of their circulating CD4 T-cells express CD28. CD28% is defined here as the proportion of circulating CD4 T-cells that also express CD28. FVC; forced vital capacity, FVC%p percentage of normal predicted FVC, FEV1/FVC; forced expiratory volume in 1 second, DLCO; diffusing capacity for carbon monoxide, DLCO%p; percentage of normal predicted DLCO. One CD28% High biopsy showed end-stage, honeycombed fibrotic lung, whereas all other histologic patterns were usual, interstitial pneumonia. *p<0.0001, **p = 0.007; †p<0.045.

### The Prognostic Value of CD28 Measures Is Independent of Pulmonary Function

We performed further analyses to ensure that the extent of CD28 expression in these subjects was an independent variable, relative to PFTs, for clinical outcome prognostications. The odds ratio (OR) for adverse clinical outcome (death or transplantation) of IPF patients with CD28% <82 was 13.0, with 95% confidence intervals (CI) 1.6-111.1. The inclusion of PFT values, as co-independent continuous variables in a logistic regression model (in addition to CD28%), did not enhance associations with adverse events, with individual OR for FVC, as a percentage of normal predicted values (FVC%p) = 1.0 (CI: 1.0 to 1.1), and for DLCO%p = 1.1 (CI: 1-to-1.2).

We hypothesized that the generally reported poor associations between single, cross-sectional PFT values and prognoses of IPF patients [37] may possibly be due, at least in part, to potential confounding effects of CD28 down-regulation. Since the mortality associated with CD28 down-regulation *per se* appears to be independent of concurrent physiologic measures, the unrecognized inclusion of patients with significant proportions of CD4^+^CD28^null^ cells in earlier analyses may have confounded assessments based solely on cross-sectional measures of pulmonary function (i.e., increase the “noise to signal ratio”). Moreover, the identification of other facile measures that are also predictive of patient outcomes, in addition to assays of circulating CD4 T-cell CD28 expressions, could be useful for clinical management, particularly if these determinations could be applicable to the IPF subjects who do not have significant CD28 down-regulation.

Accordingly, we examined the prognostic values of PFT abnormalities in the CD28% High cohort to assess whether this subpopulation could be further stratified for risks of adverse clinical outcomes (death or lung transplantation). ROC curve analyses indicated that values of FVC%p<52 and DLCO%p<38 had the best respective combinations of specificities (the most important consideration) and sensitivities for adverse clinical outcomes. Fifty (50) CD28% High IPF patients had complete pulmonary function tests (both FVC and DLCO measures) concurrent with their CD4 T-cell assays and were evaluable here. Twelve-month freedom from adverse outcomes of the CD28% High patients with FVC%p≥52 (n = 33) was 70±8%, compared to 29±11% among those with FVC%p<52 (p = 0.001). The DLCO%p seemed to provide slightly better risk stratification among the CD28% High patients, with corresponding one-year freedoms from adverse events of 76±8% and 29±10% for those with values above (n = 24) and below (n = 26) the cut-off of 38, respectively (p = 0.0003).

The potential utility of risk assessments using combinations of independent prognostic parameters may be indicated by survival analyses using *either* CD28% *or* DLCO%p as outcome indicators. Thirty-two (32) of the IPF patients studied here had either extreme CD28% down-regulation (CD28%<82) or DLCO%p values<38. Of the 11 patients that had CD28 down-regulation (Table 1), six (6) also had DLCO%p<38, another had a DLCO%p = 54, and four others did not have DLCO determinations concurrent with their CD4 assays. A total of 29 IPF patients had both CD28% ≥82 and concurrent DLCO%p≥38. Twelve-month freedom from adverse clinical outcomes was 76±8% for the latter, compared to 22±7% among those patients with either CD28 down-regulation *or* DLCO%p<38 (Figure 5B). The OR for the association between adverse clinical events and the presence of either CD28%<82 or DLCO%p<38 was 11.2, with 95% CI 3.4–37.1. The addition of low FVC%p criteria (FVC%p<52) to the combined low CD28% and/or low DLCO%p values slightly weakened the association with adverse events (OR: 10.3; CI: 3.0–34.9). Thus, the presence of *either* CD28% down-regulation or low DLCO%p values, as simultaneous, combined, cross-sectional ascertainments, would seemingly identify a large proportion of the patients within an IPF cohort who are at greatest risk for serious adverse events within the next few months.

### CD4^+^CD28^null^ T-Cells Are Present in IPF Lungs

Since the IPF subjects here were scrupulously free of extrapulmonary pathology or inflammatory foci, we hypothesized the circulating CD4^+^CD28^null^ cells of these patients originated in their diseased lungs. Moreover, the presence of these unusual T-cells in IPF lungs would further imply the associations of CD28 down-regulation with disease manifestations here (Figures 4 and 5) may not be merely epiphenomenal. Accordingly, we used confocal microscopy to examine sections of IPF lungs removed during therapeutic pulmonary transplantations.

We reasoned that tissue imaging assays based on positive expressions of specific CD4^+^CD28^null^ cell characteristics would be more reliable and preferable to finding that CD28 did not appear to be present (which could also be due to poor antibody staining). Granzyme B seemed a useful surrogate marker for this purpose, since the majority of CD4^+^CD28^null^ cells produce this cytotoxic mediator, unlike “normal” CD4^+^CD28^+^ T-cells (Figure 1B). However, since natural killer (NK) cells can also express CD4 and produce cytotoxic mediators, it was necessary to confirm the intrapulmonary CD4^+^granzyme B^+^ cells also co-expressed CD3 (and are thus T-cells).

**Figure 6:**
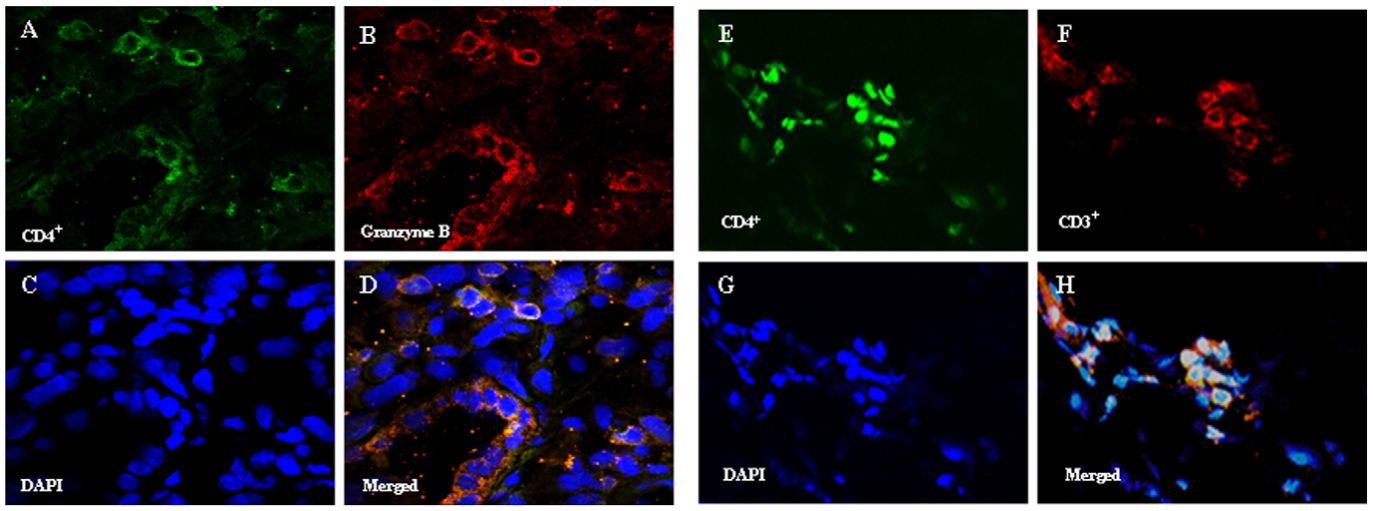
Localization of CD4, CD3, and granzyme B expressing cells in IPF lungs. *A.) and B.)* Cells expressing CD4 (Green) and granzyme B (red), respectively, are widely distributed in IPF lung sections. *C.)* DAPI (blue) stains DNA within cell nuclei. *D.)* Co-localizations of CD4 and granzyme B (yellow) among nucleated cells are seen in the merged image. Nearly all the intrapulmonary CD4^+^ cells co-expressed granzyme B. Images *E.)*, *F.)* and *G.)* similarly depict CD4 (green), CD3 (red) and DAPI (blue). *H.)* Merged image shows most of the CD4^+^ cells also co-expressed CD3. Similar results were present in both IPF lungs. The majority of CD4^+^ cells in these IPF lung sections co-expressed both granzyme B and CD3 and are, thus, CD4^+^CD28^null^ T-cells (see also Figure 1B). All images are 60x.

Confocal microscopy images revealed numerous cells positive for CD4, CD3 and granzyme B were widely distributed within alveolar epithelial areas of the IPF lungs, as well as in or proximate to fibrotic foci of both explant specimens (Figure 6).

## Discussion

These data demonstrate that the CD4^+^CD28^null^ T-cells of IPF patients exhibit unusual characteristics (Figure 1B and Figure 2) that are very similar to analogous cells isolated from other patient populations with chronic immunologic diseases [19]–[34]. Infiltrations of these unusual CD4^+^CD28^null^ T-cells were evident in IPF lungs (Figure 6). Changes of CD4 T-cell CD28 expression with replicate studies of subjects were directly correlated with corresponding changes of their lung volumes (Figure 4). Most importantly, marked CD28 down-regulation on the peripheral CD4 T-cells of individual IPF patients is associated with a higher likelihood of their requiring lung transplantations or dying during the next year (Figure 5A), and these T-cell measures also seem to complement and enhance the prognostic utility of pulmonary physiologic testing (Figure 5B).

Nearly all normal CD4 T-lymphocytes express CD28 on their cell surfaces [38], and finding significant proportions of circulating CD4^+^CD28^null^ T-cells is distinctly abnormal [19]–[34]. In all cases of which we are aware, possibly excepting the very aged, CD28 down-regulation on significant proportions of peripheral CD4 T-cells is a specific indicator of an ongoing, chronic inflammatory response, as previously described in patients with autoimmune syndromes, persistent inflammation-provoking infections, or other long-standing immunologic disorders [19]–[34].

The pathophysiologic importance of the CD28 “deficiency” *per se* in these patients is uncertain. Cognate interactions of T-cell CD28 with ligands (e.g., CD80 and CD86) provide a “second signal” for lymphocyte activation, in conjunction with the primary signals mediated by TCR engagements with antigen-MHC complexes. Nonetheless, neither previously activated (“memory”) CD4 nor naïve CD8 T-cells require CD28 for activation, and other costimulatory molecules can also provide the second signal necessary for initial activations of naïve CD4 cells [38], [39]. Since CD28 costimulation is not invariably essential to evoke T-lymphocyte responses, the absence of this molecule on CD4 T-cells of patients with immunologic diseases [19]–[34] is perhaps most immediately significant in providing a facile marker to identify the daughter progeny of repeated, antigen-driven proliferation cycles. However, certain other characteristics of the CD4^+^CD28^null^ cells, seen here in greatest proportions among the IPF patients destined for poor outcomes, may have potential pathogenic significance.

The overall cytokine production profile of the IPF CD4^+^CD28^null^ cells is highly comparable to that previously observed among analogous lymphocytes derived from distinctly different patient populations with other chronic immunologic disorders [20], [24]. Thus, it seems most likely that the singular characteristics and functional modifications of this cell phenotype are a biologically conserved (and presumably important) component of the adaptive immune response to chronic stimulation by diverse antigen(s). As such, and amongst other possibilities, the ability to generate CD4^+^CD28^null^ cells with repeated/chronic antigen engagements could conceivably represent an evolutionary adaptation to more effectively counter difficult-to-eradicate microbial pathogens [19].

Chronic adaptive immune responses in human diseases are extraordinarily complicated. Antigen-activated T-cells can, in appropriate circumstances, undergo innumerable divisions, resulting in prodigious numbers of clonal daughter progeny, including variable proportions of CD4^+^CD28^null^ cells [19]. In turn, these complex and changing subpopulations of T-cells with evolving phenotypes and functions produce diverse mediators that also activate and/or recruit successive waves of other immune effectors (macrophages, neutrophils, dendritic cells, etc.), as well as epithelium and many mesenchymal cells. These second- and third-tier effector cells also add their productions of interrelated, interactive, and often redundant mediators and other elaborations in what can become an unfathomably complex inflammatory conflagration (18).

Accordingly, the exact role(s) the singular functions of CD4^+^CD28^null^ could directly contribute to tissue injuries of IPF (or other chronic immunologic diseases [19]–[34]) *in situ* cannot be deduced from highly reductionist *in vitro* studies of single isolated cell types. Nonetheless, it may still be notable that several T_H_1 and other pro-inflammatory mediators which initiate or amplify immune responses (e.g., IL-1β, IL-6, TNF-α, G-CSF) were comparatively over-produced by the IPF CD4^+^CD28^null^ cells relative to their autologous CD4^+^CD28^+^ counterparts, both constitutively, and with TCR stimulation (Figure 2). Moreover, the perforin and granzyme B productions (Figure 1B) of the CD4^+^CD28^null^ lymphocytes could conceivably account for infiltrates of cytotoxic granule-laden lymphocytes that are present in IPF lungs [40] (Figure 6), and are believed to contribute to the pulmonary epithelial apoptosis of this disease [35]. In comparison, T_H_2 cytokine productions by the CD4^+^CD28^null^ cells were typically relatively little increased upon TCR stimulation, although their elaboration of IL-4, a potentially important mediator of fibrogenesis [41], is a notable exception.

Furthermore, the many pro-injurious mediator productions of CD4^+^CD28^null^ cells are also strikingly contrasted with their relative deficiencies of opposing, counter-regulatory functions that could potentially limit or modify inflammatory processes. The comparatively lesser production of IL-10 by CD4^+^CD28^null^ cells may have particular biologic importance in fibrotic disorders, given the possible roles of this cytokine in general suppression of deleterious immune responses [42] and inhibition of TGF-β-mediated fibrogenesis [43]. CD28 signaling has been implicated in the induction of FoxP3 and generation of CD4^+^CD25^+^ T_regs_ [44]. Consequently, lack of this costimulatory function could plausibly account for the comparative paucity of FoxP3 among the CD4^+^CD28^null^ cells, and these findings are also broadly concordant with recent observations that CD4^+^FoxP3^+^ T_regs_ are generally diminished among IPF patients (17). The net effects of the seeming imbalance between pro-injurious and counter-regulatory (dampening) functions could conceivably result in promotion of more injurious responses with serial (chronic) antigen engagements (including intrapulmonary antigens [13]) by T-lymphocyte populations that have increased proportions of CD4^+^CD28^null^ cells.

Because of the downstream amplification of T-cell effector responses [18], and extraordinary numbers of T-cells in an individual, antigen activation of even a limited fraction of total lymphocytes can have far-reaching effects. The proportion of peripheral CD4^+^CD28^null^ cells among the CD28% Low subjects here was ∼34% (Table 1). Since the circulating T-cell compartment represents only a miniscule fraction of the lymphocytes that traffic from inflamed tissue and proximate lymph nodes [45], [46], the total numbers of these highly altered lymphocytes in the IPF patients is incalculably large. By comparison, the precursor frequency for T-cells reactive to conventional peptide antigens (e.g., influenza epitopes) is estimated at 1∶1000 to 1∶10,000 [47]. Microbial superantigens are among the most potent antigens known, can mediate life-threatening toxic shock syndrome, and activate ∼−−20% of total T-cells among infected individuals [48]. Alloantigens are often highly immunogenic, capable of evoking brisk, severe, and refractory allograft rejection, and are initially recognized by ≤10% of the recipient T-cell repertoire [49]. Daughter progeny of T-cell clonal proliferations (which can develop into CD4^+^CD28^null^ cells [19]) comprise a very similar proportion of circulating CD4 T-cells (∼36%) among lung transplant recipients with severe lung damage due to obliterative bronchiolitis [50]. The frequency of CD4^+^CD28^null^ cells among the IPF patients destined for poor outcomes here are also highly comparable to the proportions of these cells among lung transplant recipients with chronic rejection who had similarly guarded prognoses [20], and is even greater than in many other disease conditions wherein these particular lymphocytes have been implicated in pathogenesis [21]–[34]. Thus, the CD4^+^CD28^null^ cells among many of the IPF patients here appear at least numerous enough to be potentially capable of causing or contributing to disease manifestations.

We do not believe the present data justify use of CD28 assays, either alone or in combinations with PFTs (e.g., DLCO%p), as a definitive clinical tool in IPF patients yet, pending additional and confirmatory study. This initial, cross-sectional, exploratory examination was focused on investigating potential immunopathogenic phenomena of IPF *per se* and, as such, we intentionally excluded subjects with infections, malignancies, accelerated IPF, or co-morbid autoimmune disorders that could, perhaps, confound the operating characteristics of these T-cell assays in actual patient management. Prospective serial observations in a large patient cohort will more accurately define the accuracy and validity of CD4 T-cell assays for clinical prognostications, and a longitudinal trial is currently in progress.

In summary, the present data show that the extent of CD28 expression on circulating CD4 T-cells of IPF patients is associated with disease outcomes. Among other implications, these findings are further evidence for a role of adaptive immune processes in the pathogenesis of IPF [3]–[15], [17]. The intrapulmonary antigen(s) that drives T-cell proliferation [13], and the eventual generation of CD4^+^CD28^null^ lymphocytes in these patients, has not yet been identified, and possibilities remain broad, including an exogenous protein(s) or hapten, persistent microbes, or even a self-epitope [3]–[7], [13]. The findings here may illuminate a novel paradigm of IPF progression that could ultimately lead to more effective counters for this devastating disease, possibly including selective targeting of singularly pathogenic lymphocyte subpopulations. If further substantiated, moreover, the practical value of simple, minimally invasive T-cell assays that identify patients destined for disease progression would be considerable. Thus, heightened surveillance and/or earlier interventions (e.g., lung transplantations or experimental therapies) could be directed to those patients at greatest immediate risk, while possibly obviating morbid treatments in those destined for more indolent courses.

## Materials and Methods

### Subjects, Assignments of Diagnoses, and Treatment Decisions

IPF patients were recruited from referrals to the interstitial lung disease (ILD) clinic at the University of Pittsburgh. Normal controls were recruited by solicitation, and did not have any known systemic or immunologic disorders that affect CD28 expression [19]. All subjects gave written informed consent *per* the University of Pittsburgh Investigational Review Board (IRB).

IPF diagnoses were prospectively established by expert, specialized ILD clinicians who analyzed all available information, including medical histories and physical exams, PFTs, laboratory studies that included serologic tests for autoimmune syndromes, chest radiographs and computerized tomography scans, and histologic evaluations of lung biopsies and pulmonary explants surgically removed during transplantations. All IPF subjects who provided blood or tissue specimens here fulfilled consensus diagnostic criteria [1]. None had ongoing acute exacerbations of IPF [3], clinical or serologic evidence of autoimmune disorders, malignancies, or concurrent infections. Subjects were also excluded if they had histologic patterns on lung biopsy (or explant) other than usual interstitial pneumonia (UIP) or, in one case, end-stage fibrotic lung disease with honeycombing [51].

The clinicians caring for these patients were blinded to the results of these experimental T-cell studies. Conversely, the T-cell assay results were analyzed by laboratory-based investigators blinded to subject identities, disease manifestations, and clinical outcomes.

### Cell Preparations and Flow Cytometry

Mononuclear cells were isolated from peripheral blood of study subjects by density gradient centrifugation for use in flow cytometry or functional assays. PBMNC for cell surface characterizations were incubated with monoclonal antibodies (MAb) immediately after their isolation, and then promptly analyzed by flow cytometry. Unless otherwise denoted, MAb, including isotype control antibodies, were purchased from BD Pharmingen (San Diego, CA). These MAb included CD4 (RPA-T4), CD28 (CD28.2), CD8 (HIT8a), MHC Class II (DR) (L243), CD25 (M-A251), granzyme B (GB11), and perforin (δG9). Anti-FoxP3 MAb (FJK-16S) was purchased from eBioscience, San Diego, CA.

Quantitations were performed on ≥10,000 live cells and analyzed using a BD FACSCalibur (BD Bioscience, San Jose, CA). This instrument has 488 nm and 635 nm lasers, and employs long pass, short pass, and bandwith filters that transmit wavelengths 530/30 nm (FL1), 585/42 nm (FL2), 670 nm (FL3), and 661/16 nm (FL4). Flow cytometry gates were set using control fluorochrome positive and negative PBMNC (including isotype controls).

**Figure 7:**
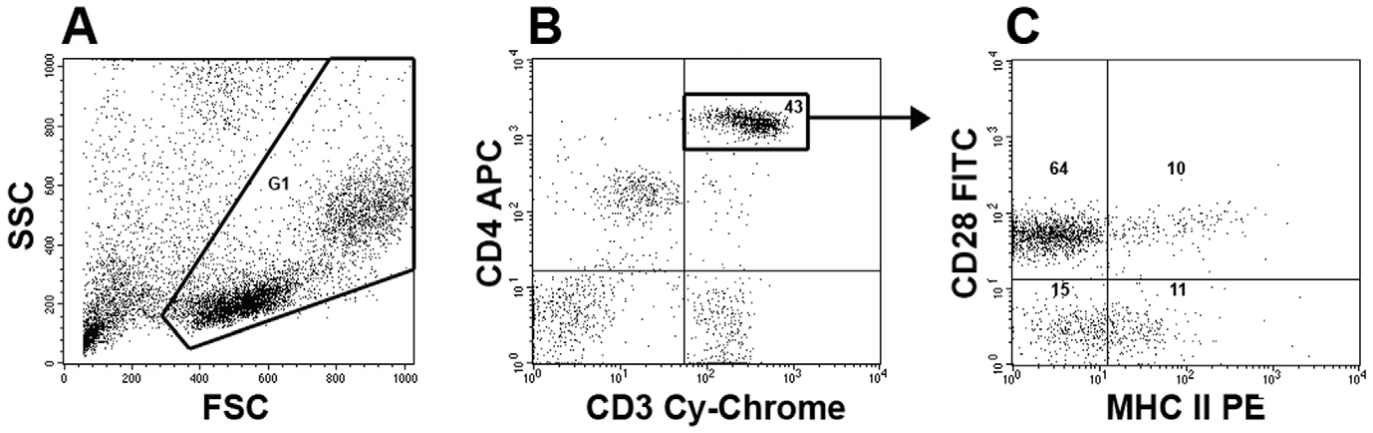
Flow cytometry methodology (see also references 20, 52). Aliquots of fresh, live peripheral blood mononuclear cells (PBMNC) were stained with anti-CD4-allophycocyanin (APC), anti-CD28-fluorescein isothiocyanate (FITC), and phycoerythrin (PE)-conjugated antibodies against other cell epitopes. *A.)* Ten thousand (10,000) or more live cells were selected for further study, based their side scatter (SSC) and forward scatter (FSC) characteristics (G1). *B.)* The brightly staining CD4 cells among these also expressed CD3 (Cy-Chrome) and, thus, are T-cells [52]. *C.)* These CD4 T-cells were further characterized based on their expression of CD28. The proportions of CD4^+^CD28^+^ T-cells among the total CD4^+^ T-cell population (upper left and upper right quadrants) defines the CD28%. The respective proportions of CD4^+^CD28^+^ and CD4^+^CD28^null^ cells that co-expressed other cell determinants of interest (in this case MHC Class II [DR]) were quantitated. Numbers within the delineated region/quadrants denote the proportions of cells with these respective characteristics.

CD28 and other phenotypic markers were quantitated within respective CD4^+^CD28^+^ and CD4^+^CD28^null^ cell subpopulations, as detailed elsewhere [20]. In brief, PBMNC were stained with anti-human CD4-allophycocyanin (APC) and anti-human CD28-fluorescein isothiocyanate (FITC) MAb, and individual aliquots of these were also stained with phycoerythrin (PE)-conjugated MAb against other cell surface epitopes of interest (e.g., HLA-DR, CD25, etc.). CD4^bright^ cells corresponding to the CD4 T-cell subpopulation were selectively gated (from within the live cell gate) for further characterizations (Figures 7A, 7B) [20], [52]. CD28 and other phenotypic markers were quantitated within respective CD4^+^CD28^+^ and CD4^+^CD28^null^ subpopulations (Figure 7C). Intracellular mediators (perforin, granzyme B, and FoxP3) were also quantitated among freshly isolated PBMNC aliquots that had been identically stained with anti-human CD4 and CD28 MAb, and then fixed and permeabilized prior to incubation with MAb having specificities for the intracellular molecules, per manufacturers' protocols.

The CD28% value for individual patients was defined as the proportion of their circulating CD4 T-cells that co-express CD28 [20]. Based on prior observations, we anticipated that study of CD4 T-cell phenotypic markers (e.g., MHC Class II, CD25, etc.) in 24 IPF subjects would provide very high power to detect intergroup differences, if their cellular characteristics resembled those of other patient populations [20]. Specimens for the T-cell phenotype studies were selected randomly, without prior knowledge of the corresponding CD28% or subject's clinical characteristics.

### Cytokine Assays

CD4 T-cells cells for cytokine and proliferative assays were isolated from PBMNC by negative selection with immunomagnetic beads (Miltenyi Biotech, Auburn, CA). CD28^null^ cells and CD28^+^ cells were segregated from among this CD4 T-cell population by staining with anti-CD28-phycoerythrin (PE), followed by positive selection of the latter with anti-PE immunomagnetic beads (Miltenyi Biotech) [20].

1×10^5^ CD4 T-cells (autologous CD28^+^ and CD28^null^) were separately cultured for two days in 96 well plates in both stimulated (10 µg/ml plate-bound anti-CD3 MAb [OKT3]) and un-stimulated (basal) conditions. Culture supernatants were analyzed for cytokine productions using a Bio-Plex™ protein suspended bead array platform (Bio-Rad, Hercules, CA), following the manufacturer's protocols. Prior observations indicated that study of six (6), randomly-selected specimens would enable detections of intergroup cytokine production differences, if characteristics of the IPF CD4 T-cell subpopulations were similar to those seen with cells isolated from another patient group [20].

### CD4 T-Cell Proliferation Assays

These methods have been described elsewhere [20]. In brief, segregated autologous CD28^+^ and CD28^null^ CD4 T-cell subpopulations, isolated by immunomagnetic beads, were individually cultured in duplicate conditions, including unmanipulated (control) incubations, and in wells previously coated with anti-CD3 MAb (see above). Autologous nurse cells, positively selected from PBMNC during the initial CD4 T-cell isolations, were added in a 3∶1 nurse cell:CD4 T-cell ratio. All CD4^+^ cells were depleted from the nurse cells, prior to their use in cultures (≪1% residual), by positive selection with anti-CD4-coated magnetic beads (Invitrogen, Carlsbad, CA).

Proliferation within the respective CD4 subpopulations were measured by bromodeoxyuridine (BrdU) incorporation, using reagents and methods supplied in a kit (BD Pharmingen). BrdU in a final concentration of 10 uM was added to cultures 2 days prior to their harvests. After 5±1 days of culture, cells were harvested from wells and stained with anti-CD4 MAb, fixed and permeabilized, and then incubated with anti-BrdU and 7-amino-actinomycin (7-AAD). The proportion of proliferating CD4^+^ T-cells (BrdU^+^) among the viable cells (≥diploid DNA content) was determined by flow cytometry.

### Lung Immunostaining

Explanted lung tissues from two IPF patients who underwent pulmonary transplantations were obtained from the University of Pittsburgh Health Sciences Tissue Bank. These tissues had been embedded in optimal cutting temperature (OCT) media immediately after explantation and stored frozen at −80°C. Lung tissues were cut into 4 micron thickness using a cryostat, and placed on slides kept at −80°C until used. These frozen sections were fixed using 2% paraformaldehyde for 20 minutes. Cell permeabilisation was carried out by using 0.1% Triton X-100 in phosphate buffered saline (PBS) for 15 minutes. Sections were rehydrated by three cycles of incubation for five minutes each in PBS, followed by washing with 0.5% bovine serum albumin (BSA) in PBS. The slides were then blocked with 5% donkey serum (Sigma-Aldrich, St. Louis, MO) in PBS containing 3% BSA for 45 minutes. Slides were incubated overnight at 4°C in blocking solution supplemented with rabbit polyclonal anti-human CD3 (ab5690, Abcam, Cambridge, MA), and mouse anti-human CD4 MAb (SK3, BD Biosciences). Separate sections were similarly prepared with rabbit polyclonal anti-human granzyme B (ab4059, Abcam) and SK3 MAb. Sections were washed 5 times with 0.5% BSA in PBS to remove unbound primary antibodies, and then incubated with Alexia 568-conjugated goat anti-rabbit and Alexia 488-conjugated goat anti-mouse secondary antibodies (both from Invitrogen) for one hour at 28°C. All primary and secondary antibodies were used at 1∶100 dilutions. Subsequently, the slides were washed five times with 0.5% BSA in PBS, followed by five washes with PBS alone, with each wash lasting for five minutes. Cell nuclei were counterstained with 4′,6-diamidino-2-phenylindole (DAPI) (Vector Lab, Burlingame, CA) for one minute and briefly washed two times in PBS. Slides were then cleaned and cover slips were mounted using Gelvatol media obtained from the Center of Biological Imaging at the University of Pittsburgh. The slides were dried overnight at 4°C while protected from light, and images were visualized using an Fluorview 1000™ confocal microscope (Olympus, Center Valley, PA). Digital image data were archived and prepared for publication using Adobe Photoshop software (Adobe Systems Inc., Mountain View, CA).

### Statistical Analyses

Intergroup comparisons of continuous data were analyzed by Mann-Whitney rank-sum tests. Dichotomous variables were analyzed by Chi-square. Correlations between continuous variables were established by Spearman rank order. Survival analyses were performed using product-limit estimation, with comparisons by Log-rank (Mantel-Cox). Odds ratios and confidence intervals were established by logistic regression. Data were analyzed using StatView™ (SAS, Cary, NC.) Receiver operator characteristic (ROC) analyses were examined using Analyse-it™ v 2.12 (Analyse-it Software, Ltd, Leeds, UK). Significance was defined as p<0.05. Data are depicted as mean ± SEM.
